# Oncology Patients’ Experiences With Novel Electronic Patient Portals to Support Care and Treatment: Qualitative Study With Early Users and Nonusers of Portals in Alberta, Canada

**DOI:** 10.2196/32609

**Published:** 2021-11-24

**Authors:** Amanda D Santos, Vera Caine, Paula J Robson, Linda Watson, Jacob C Easaw, Olga Petrovskaya

**Affiliations:** 1 Faculty of Nursing University of Alberta Edmonton, AB Canada; 2 Cancer Care Alberta Alberta Health Services Edmonton, AB Canada; 3 School of Public Health University of Alberta Edmonton, AB Canada; 4 Faculty of Nursing University of Calgary Calgary, AB Canada; 5 Faculty of Medicine Department of Medical Oncology University of Alberta Edmonton, AB Canada; 6 Cross Cancer Institute Alberta Health Services Edmonton, AB Canada; 7 School of Nursing University of Victoria Victoria, BC Canada

**Keywords:** patient portal, MyChart, health information and communication technology, eHealth, personal health information, oncology, cancer care, Canada, qualitative, context of technology implementation

## Abstract

**Background:**

With the current proliferation of clinical information technologies internationally, patient portals are increasingly being adopted in health care. Research, conducted mostly in the United States, shows that oncology patients have a keen interest in portals to gain access to and track comprehensive personal health information. In Canada, patient portals are relatively new and research into their use and effects is currently emerging. There is a need to understand oncology patients’ experiences of using eHealth tools and to ground these experiences in local sociopolitical contexts of technology implementation, while seeking to devise strategies to enhance portal benefits.

**Objective:**

The purpose of this study was to explore the experiences of oncology patients and their family caregivers when using electronic patient portals to support their health care needs. We focused on how Alberta’s unique, 2-portal context shapes experiences of early portal adopters and nonadopters, in anticipation of a province-wide rollout of a clinical information system in oncology facilities.

**Methods:**

This qualitative descriptive study employed individual semistructured interviews and demographic surveys with 11 participants. Interviews were audio-recorded and transcribed verbatim. Data were analyzed thematically. The study was approved by the University of Alberta Human Research Ethics Board.

**Results:**

Participants currently living with nonactive cancer discussed an online patient portal as one among many tools (including the internet, phone, videoconferencing, print-out reports) available to make sense of their diagnosis and treatment, maintain connections with health care providers, and engage with information. In the Fall of 2020, most participants had access to 1 of 2 of Alberta’s patient portals and identified ways in which this portal was supportive (or not) of their ongoing health care needs. Four major themes, reflecting the participants’ broader concerns within which the portal use was occurring, were generated from the data: (1) experiencing doubt and the desire for transparency; (2) seeking to become an informed and active member of the health care team; (3) encountering complexity; and (4) emphasizing the importance of the patient–provider relationship.

**Conclusions:**

Although people diagnosed with cancer and their family caregivers considered an online patient portal as beneficial, they identified several areas that limit how portals support their oncology care. Providers of health care portals are invited to recognize these limitations and work toward addressing them.

## Introduction

### Background

Clinical information technologies and consumer eHealth tools are becoming an essential part of health care delivery. Patients are eager to have electronic access to their personal health information, and expectations to manage their own health have increased [1]. eHealth refers to the application of digital health technologies and includes telehealth and remote monitoring, the use of mobile devices, ePrescribing, health information technology systems, electronic health records, and more [2]. The use of eHealth and the internet has the potential to augment health care services by educating and empowering patients, making health care more equitable by extending services, expediting access to medical information, and ensuring the information provided is evidence based [3]. Furthermore, eHealth is transforming the way patients and providers communicate, establish rapport, and receive care, as virtual medical appointments become more commonplace (a movement catalyzed as a result of the COVID-19 pandemic) and as patients have immediate access to medical information.

Patient portals are secure computerized applications that give citizens access to some of their personal health information stored in health providers’ electronic health record, via electronic devices such as computers, cellphones, and tablets. Personal health information available via portals typically includes laboratory results, medications, immunizations, allergies, diagnostic results, and medical visit notes [4]. Other portal features include secure messaging with health care providers, appointment self-scheduling, and requesting medication refills. Different jurisdictions may choose to enable different portal features and set restrictions (ie, immediate test result release versus embargo period).

In addition to portals, digital platforms, including the internet, enable access to health-related information and peer support groups. The internet is often used as a primary source of health-related information, generating concerns about misinformation among health care providers [1]. To address this concern, patient portals that provide hyperlinks to credible information (eg, medication side effects, explanation of laboratory tests) have been suggested as a preferred source of information.

In Canada, patient portals are becoming more available, but the actual use is challenging to estimate. In Alberta, a Canadian province with a population of more than 4.4 million people, 2 province-wide patient portals were launched in 2019: MyHealth Records and MyAHS Connect (MAC; described later in the paper). As of March 31, 2021, approximately 565,000 Albertans had created a MyHealth Records account and more than 38,000 Albertans had access to the MyAHS Connect portal [5]. The latter figure denotes the total number of patients who either started using or could *potentially* start using MyAHS Connect as this portal was gradually becoming available across the health care sites these patients visited.

The patient portal use in Alberta may not necessarily be representative of the overall population in Canada. For example, in 2019, the Canadian Medical Association reported that virtual care and online patient portals were used by 1% of Canadians [6]. The same year, researchers at the University Health Network (UHN) in Toronto, Canada, reported the annual adoption rate of approximately 65%, with 43,000 “myUHN” patient portal registrations during the first 14 months [7]. Attempts to reconcile these numbers should be made with caution. On the one hand, reports of portal adoption are often based on a nonconservative definition of portal use, meaning activating a portal account or logging in once. On the other hand, all sources from 2019 cited above reflect a pre-COVID-19 pandemic situation. During the pandemic, virtual care and portal adoption have been on the rise.

Canadian research to demonstrate the impact of patient portals is emerging. Similar to international studies, Canadian research suggests that there are benefits to using portals [1]. Patients often value portals, as this technology provides them with detailed information about their health and stimulates and informs conversations with their health care providers [8,9]. Furthermore, being able to schedule appointments, request medication prescription renewals, and access medical information allow patients to feel more involved in the management of care [9]. Health care providers comment that portals give patients the opportunity to actively participate in the management of their care and that patients are better prepared for medical appointments, as they have additional time to look up medical results and develop pertinent questions [9]. Portals may also benefit health care systems, as patients might be more willing to follow medical advice and more diligent with refilling medication prescriptions [9].

Despite these benefits, there are barriers to portal implementation and use. Limited health and digital literacy and lack of computer or internet access increase health inequities and further marginalize selected population groups [10]. Test results may be misinterpreted by patients, generating anxiety and increasing the demand on health care professionals to provide reassurance and clarification to their patients [11]. In addition, health care organizations have reported concerns regarding limited financial resources to implement patient portals [12].

Patient portal use is known to be the highest among patients diagnosed with cancer. The Canadian Cancer Society [13] predicts that approximately 1 in 2 Canadians will be diagnosed with cancer in their lifetime, and about 1 in 4 will die of the disease. With the steady year-on-year increase in cancer diagnoses, online patient portals are becoming more desirable to augment the coordination of care for oncology patients [14]. Cancer treatment and the cancer diagnosis, in and of itself, result in a wide range of self-management challenges, such as monitoring side effects and scheduling numerous medical appointments. Oncology patients have a keen interest in portals, as they require comprehensive health information, have blood work done regularly, and often are, or are expected to be, active participants in managing their condition [14,15]. They report that using portals allows them to feel more in control of their situation, be better prepared for medical appointments, and provides them with the opportunity to advocate for their needs [16]. Yet, some oncology patients view portals with reservations. For example, with the immediate release of laboratory and imaging results via a portal, patients may discover that their cancer has metastasized. Given the implications of living with cancer, oncology patients are often viewing these results during times of despair, thereby compounding feelings of fear and uncertainty [17].

The objective of this study was to explore how patients diagnosed with cancer use online resources for care and treatment in the Canadian province of Alberta. Specifically, we were interested in patients’ awareness and use of the novel electronic patient portals in Alberta’s unique, 2-portal context.

### The Context and Setting of This Study

When reporting research on patient portals, it is important to clearly outline characteristics and functions of specific portals and describe sociopolitical and organizational contexts of portal implementation and utilization [18]. Below we describe the complicated context of portal implementation in Alberta, Canada, where this study was performed.

In March 2019, Alberta’s Ministry of Health (Alberta Health) released a provincial patient portal called MyHealth Records (later, its component called My Personal Records [MPR] became a patient portal per se) allowing all Albertans 14 years and older to access some of their health information online, most notably immunization records and common laboratory results [19]. MyHealth Records requires a multistep process to create an account and authenticate (as described in detail below). All patient information is supplied to MyHealth Records from a provincial electronic medical record (EMR) called Netcare. Although useful to health care providers, Netcare EMR is a “view-only” system.

In November 2019, Alberta Health Services (AHS), the province’s integrated health authority, launched Wave One of the clinical information system, Connect Care (AHS’ name for its project to implement the EPIC system), in some acute care teaching hospitals and ambulatory clinics in Edmonton. Connect Care implementation is an ongoing ambitious process consisting of 9 waves (from 2019 to 2023), with 3 waves already launched, aiming to achieve the *one patient one record* goal for the province. Unlike clinical information systems implemented in a single health care facility or across a few facilities, Connect Care is envisioned to span the entire province with the population of more than 4.4 million people and to replace existing fragmented EMRs. One of the future waves will include oncology facilities across the province. As a component of Connect Care, AHS offers a tethered patient portal called MyAHS Connect (MAC; known as MyChart during the pilot stage, as described below) to enable patients registered with AHS facilities to access their personal health information [20].

In preparation for Connect Care launch, from 2015 to 2019, the AHS piloted its tethered patient portal called MyChart (EPIC) in select Edmonton clinics. Patients who used MyChart during the pilot stage were mostly satisfied with the portal and described it as an easy-to-use, efficient tool that improved accuracy of data sharing and allowed for easier communication [8,21]. Although a sign-up process presented initial challenges for some patients, overall, it was easy to create a MyChart account, including obtaining proxy access. With Wave One of Connect Care in 2019, AHS initially made an arrangement for existing MyChart users to be “grandparented” into the new, Connect Care–enabled patient portal. However, due to the tensions between the 2 macro-level portal implementers, access to the portal for these existing users was interrupted either temporarily (they had to create a new account) or permanently (for some parent proxies who accessed their children’s information). In early 2020, MyChart was renamed MyAHS Connect and the access to this portal was streamlined with the Government’s MyHealth Records patient portal, which affected the ease of enrollment for AHS patients, as described below. A chronology of major events in Alberta, up to April 2019, leading to the unique, 2-portal context in the province is presented in Avdagovska et al [22].

Thus, at the time of our study in the Fall of 2020, Albertans who were patients attending AHS facilities could enroll to view their personal health information via one or both online portals accessible through the Government of Alberta website under the aegis of MyHealth Records: (1) a provincial citizen portal My Personal Records (MPR) linked with a “view-only,” legacy EMR; and (2) the MyAHS Connect (MAC) portal tethered to a Connect Care–enabled EMR. (Refer to Multimedia Appendix 1 for a table comparing portal features in Alberta. Portal functionalities are categorized based on Ammenwerth et al [23] with adaptations).

To sign up for MyHealth Records, citizens must access the Government of Alberta website, register for a MyAlberta Digital ID (MADI), and confirm their identity by uploading an Alberta driver’s license or Alberta ID card. Within 10 days, one receives a verification code in the mail and is able to complete MADI registration online. A person then has to provide his/her personal health number (each legal resident has this number to access the Canadian publicly funded health care system) to set up access to the My Personal Records portal. To access the MyAHS Connect portal, in addition to the above steps, a patient must be attending an AHS health care facility that has launched Connect Care, and be offered or indicate their interest in becoming a portal user to the facility’s personnel, who will provide further instructions (ie, a website link to enter personal information to get access to MyAHS Connect) [24].

Of note, AHS facilities, in which Connect Care is being implemented, include hospitals, outpatient clinics, continuing care facilities, cancer centers, mental health facilities, and some community health sites across the province. By contrast, some primary and community care sites, and family physicians are not officially part of AHS and additional efforts will be required to link these sites to Connect Care.

As is evident from the above description, for the public, major challenges in accessing Alberta’s portals include a complicated sign-up process, terminological confusion with many variants of official and colloquially used portal names and abbreviations, additional steps for proxy access for parents of sick children (as children under 14 years of age cannot have a MADI account), and what appears as the existence of 2 parallel portals.

Apart from a few studies conducted during the MyChart pilot stage [8,21], there is limited understanding of the use and effects of patient portals in Alberta. The research question guiding this study focused on patients diagnosed with cancer to explore their experiences of using online resources to support their cancer treatment and care, and in particular patients’ awareness and use of the novel electronic patient portals in Alberta, Canada. We sought to understand how Alberta’s unique, 2-portal context shapes experiences of early portal adopters and nonadopters, in anticipation of a province-wide rollout of a clinical information system in oncology facilities.

## Methods

### Design

This qualitative descriptive study [25] involved in-depth semistructured interviews with oncology patients and their family caregivers to provide a comprehensive summary of the phenomenon under study. Broadly, our theoretical assumptions informing the study relate to the *technology-in-practice*, *sociomaterial* perspective [26,27]. This perspective conceptualizes technologies as active artifacts whose role and effects can be better understood in their relation to other human and nonhuman actors in a person’s situated reality. The technology-in-practice perspective helps to avoid both the uncritically enthusiastic rhetoric of technological progress as always beneficial and an equally unwarranted negative technological determinism (eg, cold technology eliminates warm human touch). Rather, a researcher is guided to study how technological objects are used or not used in everyday life *in connection* with other human and nonhuman actors; what human actors do with those objects; and what those objects *do*, what effects they produce. Ethical approval for this study was obtained from the Research Ethics Board at the University of Alberta (Pro00098299).

### Sample and Recruitment Strategy

Using convenience, purposive sampling, we recruited 11 participants who had been previously or were presently diagnosed with cancer or their family caregivers, were residents of Alberta, and spoke English. Our primary interest was the experiences of patients diagnosed with cancer. However, it is well known that in the context of oncology care, family involvement (eg, informal and unpaid caregiving provided typically by close family members) can be significant. Thus, we reasoned that eligibility criteria inclusive of family members of people diagnosed with cancer may attract more than 1 person from the same family unit. For instance, a patient in an active stage of cancer might choose to participate with the assistance of a family member. As described below, only 1 participant in our sample self-identified as not diagnosed with cancer but as a family caregiver with past experiences of caregiving, and rather than excluding this person, we interviewed him and clearly marked his data in the findings as provided by a family caregiver.

A recruitment email was sent twice, 1 month apart, to more than 100 members of the Cancer Care Alberta Patient and Family Advisory Network. This Network is a group of volunteers, often retired professionals, actively interested in providing their opinion to AHS on various health-service related topics. We reasoned that the Network is a group of accessible informants with direct experience with cancer, who moreover are likely to be aware about the novel patient portals. The portals have not been widely advertised in the province, and thus we targeted a group that is generally more informed about health service innovations in Alberta. Interested individuals contacted the lead author (ADS) directly over email or phone to schedule the initial consent meeting. All 11 respondents who took part in the individual consent meetings agreed to participate in the study.

### Data Collection

From August to November 2020, each participant completed an online demographic survey and took part in a semistructured interview over the phone. We developed the interview guide to be aligned with the technology-in-practice perspective. That is, rather than asking participants who self-identified as portal users to explain how the portal is helpful and why it is good, we asked a broad opening question about using (or not) online tools and resources while living with a cancer diagnosis. We further asked participants to describe situations in which they used the internet or the portal, for example, “What happened that you needed to use an online tool?,” “What did you look for?,” “How did you use the information?.” An interview guide was used to evoke detailed responses from all participants [28]. Interviews ranged from 27 to 68 minutes in length, with an average time of 48 minutes, and were audio-recorded and transcribed verbatim. During the interviews, the interviewer (ADS) took reflective notes to enhance credibility and trustworthiness of the study, as personal beliefs and preconceived notions were brought forth [29]. The interviewer did not know and had no interaction with the participants prior to the study.

### Data Analysis and Rigor

An inductive thematic analysis was undertaken [30,31]. Transcripts were coded by the lead author. All codes and associated quotes were compared and contrasted to identify similarities and differences across the data set. Codes were then grouped into preliminary categories and themes, and were finalized once all codes and preliminary categories were reviewed and discussed with 2 other members of the research team (VC and OP) until a consensus was achieved, ensuring the qualitative rigor of the study [32]. Data analysis occurred simultaneously with data collection until no new codes were identified.

Saturation, or the point in the data collection process when participants provide similar information [33], was reached at diverse points for different themes. For example, by the fifth interview all participants talked about the uncertain future they face once diagnosed with cancer and how they searched the internet for health-related information and how they desired transparency when communicating with health care providers. These ideas are expressed in what we identified as Theme 1. By the ninth interview we had consistently heard that most portal users were trying to gain independence by being able to access information via a portal, using the portal to prepare for appointments, and disliking incomplete information and poor organization of the portal webpages. This too shaped subsequent themes.

One of the trustworthiness criteria in qualitative research relates to the expertise and experiences of researchers [33]. To present a compelling account of the phenomenon under study, researchers need to strike a balance between possessing knowledge of the field of study (eg, to create data collection tools, understand the context) and delineating between their own assumptions and participants’ experiences. Our research team brought relevant expertise and self-awareness to this study: one of the members of the research team had received cancer care recently, adding an important patient perspective during team discussions. Another academic member of the research team (OP) focuses on eHealth and portal technology implementation, contributing expertise in this area. Authors from Cancer Care Alberta (AHS) include a member of the Executive Leadership Team (PJR), a scientist (LW), and an oncologist (JCE), each of whom have interests and experience in exploring innovations in models of cancer care.

## Results

### Participant Characteristics

Participants included 8 females and 3 males within the age range from mid-20s to late-70s. Most participants were aged 60 and above. Except for 1 family caregiver, all of the participants had been diagnosed with some form of blood-borne, tissue, organ, or lymphatic cancer. All participants reported level of education above high school, with 6 possessing university degrees. Nearly half of the participants had previously worked or were currently working in health care. All participants spoke English as their primary language, and 9 self-identified as white. All participants classified themselves as proficient users of computers, who employ internet daily for a variety of purposes such as emailing, online banking, shopping, and health information seeking.

Seven participants were enrolled in and used a portal: 1 person used both My Personal Records (MPR) and MyAHS Connect (MAC); 5 used My Personal Records only, as MyAHS Connect was not launched at their health care facilities yet; and 1 person used MyChart in the past (precursor to MyAHS Connect) and was in the process of creating her MyHealth Records/My Personal Records account.

Only 2 of 7 portal users originally learned about the portals from the public sources such as newspapers and media, whereas the majority learned about the portals from volunteering on the patient advisory committees for health services. Three participants were not aware of the portal(s) prior to the study. The only participant who did not sign up for a My Personal Records provincial portal despite being aware about it had frequent follow-up meetings with his oncologist where blood work was reviewed, which seemed sufficient in terms of accessing personal health information for this participant.

At the time of this study, all participants experienced relatively stable health (ie, active cancer treatments were completed), and used the portals from a couple of times per month to once every few months. Four participants reported having other chronic conditions, which also motivated some of them to use a portal regularly.

In the interviews, participants discussed an online patient portal as one among many tools (including the internet, phone, videoconferencing/telemedicine, print-out reports) available to make sense of cancer diagnosis, treatment, and prognosis; maintain connection with health care providers; and interact with the information. Thematic analysis of interview transcripts generated 4 key themes *reflecting the participants’ broader concerns within which the portal use was situated*: (1) experiencing doubt and the desire for transparency, (2) seeking to become an informed and active member of the health care team, (3) encountering complexity, and (4) emphasizing the importance of the patient–provider relationship.

### Theme 1: Experiencing Doubt and the Desire for Transparency

#### Overview

Several participants described using portals and the internet to reveal what they believed was the “hidden truth” about their condition. Experiencing doubt and the desire for transparency were articulated through the following subthemes: an uncertain future and transparency of health information versus withholding information.

#### Subtheme 1A: The Uncertain Future

Many participants voiced their concerns about not knowing what their future held. They used a patient portal and the internet to look for certainty. For example, when participants were asked what one was looking for or hoping to achieve while using the internet, a family caregiver replied, “My uncle I think was just wanting to know what other people had to say, what was the collective wisdom on this...am I gonna survive it?” Similarly, a woman in her 20s said the following about accessing information on social media:

There’s just so many people out there like you and sometimes it inspires a sense of hope, these people survived, I can do it too type of thing, but other times...it can cause some harm because if you see a really sad story, you’re like shoot, what if that happens to me?

Most participants found patient portals useful for accessing personal medical information, particularly test results. The words of a 60-year-old woman who used My Personal Records (MPR) exemplify an attitude of several participants: “The way I’m wired, I freeze if I don’t know the information; I freeze. Information keeps me moving forward...[this] is the best way to summarize how I use the portal, and how I use the internet.” To clarify medical terminology encountered in the portal and to search for additional information, all participants commonly turned to the internet (eg, the Mayo Clinic and WebMD websites). Participants’ preferences varied: some used Wikipedia as a starting place and then triangulated information from various sources; others sought out open access scientific research.

However, participants realized that neither generic nor personal medical information such as test result numbers in the portal provide definitive answers or allow them to understand the prognosis of their illness. For this, participants relied on their health care providers and were very sensitive to what their providers disclosed and withheld.

#### Subtheme 1B: Transparency of Health Information Versus Withholding Information

Access to medical information via a portal addressed only a fraction of what participants living with cancer felt was necessary for them. Participants often equated transparency of information with openness of their health care providers. The majority of the participants stressed the importance of receiving clear and unambiguous health information. A 64-year-old woman emphasized this notion by saying:

When you’ve got an oncology patient, for the most part,...those people really have to buy-in to the health care system, they’re there for a long time, not a good time, and they want full knowledge, they want to be able to get confirmed...what’s the word I want...full consent, knowledgeable consent.

Comparably, a 68-year-old woman disclosed how she used nonverbal cues to attain openness during a telehealth videoconference: “When I asked him [oncologist] a question, I could look to see if he was covering anything, you know, if he was trying to protect me from some information, I could tell that on his face.” (This video call was enabled by other technologies, not via the portals, as My Personal Records does not provide video visits with health care providers). Participants implied that honesty and full transparency are inextricably intertwined; both are paramount to the provision of care and to the development of trustworthy patient–provider relationships. As a 72-year-old man stated:

We don’t want secrecy, we want openness. The health system is all about the patients and without the patients you don’t have a business....If you’ve got an open thing of information on both sides of the conversation, you can overcome objections so much more honestly.

It is noteworthy that many participants wondered if their health care provider was withholding information from them as a means of protection. A 45-year-old woman said, “Because you know, you always think that maybe, are they [health care providers] telling you everything? Are they hiding something?” As a result, some participants relied on the portal and other online sources, such as social forums and websites that provide cancer-specific information, to uncover the “hidden truth.” A 60-year-old man used the internet to verify if the information he was given by his doctors was true:

I was getting statistics on the type of treatment that I was going to get and it had a success rate of well over 90% and sometimes it’s the old saying, that if it sounds too good to be true than it probably is, well I guess I checked it [the internet] to cross reference that and to make sure that they are telling me the truth about it.

Another 60-year-old participant echoed the aforementioned concern and described how she used My Personal Records to cross-check the information she received from her doctor:

You [the patient] do get left behind and I think what the portal can do...is make sure I’m asking the right questions, like why is that high and [the doctor is] not mentioning it?...to say I don’t trust the system is too extreme, but I don’t trust that people don’t make mistakes.

Most of the participants acknowledged the importance of having truthful information, often obtained from a combination of sources that assisted them during decision-making processes.

### Theme 2: Seeking to Become an Informed and Active Member of the Health Care Team

#### Overview

Much noted benefits of patient portals were having access to laboratory test results and a medication list. Participants wanted to use portals to become well-informed and better prepared for medical appointments with their oncologists; however, they felt that having access to limited information supplied via the portal prevented this from occurring. Although the portal allowed participants to feel more in control of their situation, it did not necessarily equip them to be full participants in their care because of limited information provided in the portal. Subthemes for this category included seeking control through independence, accountability for managing one’s health, and preparation for medical appointments.

#### Subtheme 2A: Seeking Control Through Independence

Prior to the adoption of portals, participants received relevant personal health information entirely through their health care providers. Portals allowed them to access test results independently and thereby made them feel more in control of their situation. A 64-year-old woman who used My Personal Records (MPR) said,

Until some of these portals were coming up, I kept a written log, I asked for copies of lab results, especially when they were abnormal. And that’s not necessary now, it’s all there online, and it is fully accessible in Alberta. [She continued] I guess it [a portal] just gives you a sense of control which I think, when you’re a patient you often feel like you don’t have a lot, so even just giving you that sense so you really felt like you were part of the team.

Reiterating this point, another participant familiar with My Personal Records, who in the past was a family caregiver, spoke hypothetically about how portals might be helpful for oncology patients:

Portals would help them [family/friends with cancer] feel more in control of what can sometimes feel like a situation where you don’t have any control. Cause you know, you’re always waiting for somebody else to tell you what’s next, and how this is gonna go, was your scan clear, was there something on it? You can go and check them yourself.

#### Subtheme 2B: Accountability in Relation to Managing One’s Health

Many participants believed that being a self-advocate and taking ownership for their own health was part of their responsibility as a patient. An online patient portal both required and promoted self-responsibility. A 64-year-old woman said,

One of the things I have found dealing with long-term residual results from cancer treatment is: if you’re not your own advocate, if you don’t stay on top of it yourself, then ...you can get lost in the shuffle. And so, to me, there is a personal responsibility for keeping on top of everything.

Although all 6 of the participants who accessed My Personal Records appreciated having the ability to independently look up their laboratory results and immunization records, many found it particularly challenging to track their health status, as the information provided to them within the portal was fragmented. A 68-year-old woman said with irony in her voice: “We want people to take responsibility for their own health and yet we are not giving them all the information.” Many participants wanted to be able to read unredacted clinic-visit summaries, doctor’s notes, referrals, and diagnostic results in full detail—regardless of how harsh those details were. However, at the time of the study, the amount of information supplied to the My Personal Records patient portal from Alberta’s EMR was very limited.

A man in his 70s shared that one of the reasons he did not access this portal was because of missing information (at the time of the study in the Fall of 2020): “PSA [prostate-specific antigen] is not available and for prostate cancer people that are in active treatment the first thing that the patient will look at is, what’s my PSA?” By contrast, a woman who had access to both portals appreciated viewing diagnostic imaging reports such as scans and X-rays provided by MyAHS Connect (whereas they were unavailable in My Personal Records). This participant found that printing out her imaging report for a muscular-skeletal injury she had been dealing with recently, and taking the report to her physiotherapist, made communication easier for her with her care provider. It also increased the accuracy of information conveyed.

Many of the participants recognized inequality in the distribution of health information. A 68-year-old woman stressed: “If we really think patients are part of the health care team then we need to give them the same information as the other members.” Being their own advocate and having equal access to medical information were considered essential components in terms of managing one’s health. Yet, most of the participants felt that My Personal Records, in its current form, was “lacking in execution.”

#### Subtheme 2C: Preparation for Medical Appointments

Given the time constraints of medical appointments with oncologists, participants really valued their appointments. For example, a 64-year-old woman said: “[A portal] allows me to be more knowledgeable when I go into a meeting or an appointment because I have specific pinpoint questions, so that I’m not wasting their [oncologists’] time.” Many participants used the portal and other online sources as a means of preparing for their appointments. A 74-year-old man shared his perception of the internet’s potential: “It is all intended to help the individual become more conscious of their situation...so that they can be more effective in their dialogue with their oncologist.” The portal and internet sites allowed participants to assume a more active role during their appointments, as having access to information prior to the meeting fostered meaningful dialogue with their oncologist. A 45-year-old woman discussed how she used My Personal Records to prepare for her appointments, “When you go see an oncologist the time is very short....So, if I go in and I already know, ok my test results were good, then my set of questions are gonna be this.”

By contrast, some participants felt that the information provided to them via My Personal Records neither prepared them for their appointments nor promoted conversations within the multidisciplinary health care team. For example, a 39-year-old woman disclosed that having access to incomplete information did not increase her confidence going into an appointment:

It [the portal] didn’t really give me that ability to come into the appointment ready, which is what I would want out of this, is for me to come into appointments more knowledgeable, for me to be able to talk with my doctor more back and forth versus him coming in with all the information.

### Theme 3: Encountering Complexity

#### Overview

All participants encountered multiple complexities when navigating the portal technology and when piecing together information. Because of the difficulty of comprehending medical jargon and unexplained information in the My Personal Records (MPR) portal, all 6 participants who used this portal turned to the internet to gather information about their medical condition. During the interviews, it was apparent how challenging the portal names were for participants, not to mention the fact that there are 2 different portals housed on the same My Health Alberta Government website. One woman felt exasperated trying to make sense of all the names, official and colloquial, she previously heard as being used (often interchangeably) to refer to a website with patient’s health information: “my health Alberta; my health; my health records; my personal records; mhr; portal; my ahs connect; my ahs; mac...” And this list does not include a mobile app version for MyAHS Connect called “MyChart by Epic.” A sense of encountering complexity and feeling lost were expressed through the following subthemes: a counterintuitive tool and difficulties comprehending information.

#### Subtheme 3A: A Counterintuitive Tool

The majority of the participants who accessed My Personal Records discussed diverse difficulties they experienced, such as poor organization of the webpage and nonintuitive navigation. A 39-year-old woman, who reported using the portal since early 2019 when it was launched, described it as “not patient-friendly.” She elaborated by describing the layout of the page with medication prescriptions: “It had dates, but it didn’t really seem like they were in order or I couldn’t really determine what the order was supposed to be, it didn’t really make sense.” Similar problems were reported by a 60-year-old woman: “Occasionally I want to check [my medications], especially the one-off prescriptions, the ones you have to spend hours digging through the data to find out what you were prescribed, like when I had a bladder infection.”

The way laboratory results were displayed in My Personal Records garnered even stronger criticism: “It just sucks,” mentioned a participant and then elaborated:

You can’t just pick a test and then get the entire bit of information...Like my mom is following her one blood test every month...If she wants to track how that one test is doing, she has to keep a written log because otherwise she has to keep going back and searching, and searching through all of the multiple blood tests she gets...I think it [My Personal Records] was designed by a computer programmer who didn’t understand how people used their data.

Similarly, a 68-year-old woman, who used to work in health care and self-identified as highly computer literate, described her attempt to make sense of the laboratory results page: “You can’t just look at it and see it on one page; that really frustrates me. And if I recall correctly, it’s organized in a weird way.”

Because of the perception of poor organization of the webpage and its “cluttered” interface, participants described the portal as difficult to navigate. A 68-year-old woman quoted above, summed up her frustration: “There’s too much stuff on it and so you have to kind of figure things out.” She continued, “[Unlike MPR] I like nice, simple, clean...here’s what I’m looking for, click on that, ok there it is.” Navigating the complicated interface deterred a 39-year-old woman from using My Personal Records: “I found it pretty hard to navigate...I just didn’t find it helpful, near as helpful as I expected it to be or hoped it would be, so I haven’t really gone back.”

In addition, participants described the multistep sign-up process as being somewhat “cumbersome.” Waiting for a code to arrive in the email felt to some like a “drag.” Further, a 45-year-old woman shared:

I had trouble signing in when [the portal became available] because you were supposed to scan your driver’s license or something, I don’t know, something wasn’t working so I actually had to try about three or four times.

Although most of the participants felt that the sign-up process was disconcerting, many appreciated, from a security standpoint, how careful the Government was at protecting information. As one person expressed, “It was worth it to go through the steps to know it was secure.”

Only 3 participants considered My Personal Records as “easy to navigate” (1 of these individuals was also referring to MyAHS Connect), while others expressed the need for a simpler portal. “The biggest thing is that they’re [portals should be] intuitive.” Another individual said, “They [should not] be difficult, portals are only as good as they’ve been created and set up and if it’s difficult to maneuver through it, it’s gonna turn people off.”

#### Subtheme 3B: Difficulties Comprehending Information

All 7 participants who used a portal encountered unfamiliar medical terminology or incomplete information and relied on the internet at some point to fill the gap. A 77-year-old woman, who previously worked as a health care provider, described having difficulty interpreting radiology reports within MyAHS Connect: “Some of these radiology words are a bit challenging and I’ve got a health care background, so if I can’t figure it out, what about the general public?” Comparably, a 39-year-old woman said, referring to a disjuncture between vaccine’s names commonly used in colloquial language and vaccine’s scientific names used in the portal: “I didn’t know...the technical name of the immunization...was that flu shot, was that Twinrix, was that the things that we call them, the layman’s terms. It was...too technical for a patient, it wasn’t patient friendly.” The same participant, who self-identified as health literate and computer-savvy and came across as very articulate, nevertheless mentioned the following about laboratory results: “It would be great if I could see all of them, or if I could understand them.”

As a consequence of encountering medical jargon and incomplete information, participants either gravitated toward the internet to understand the information or turned away from the portal altogether. For example, a 68-year-old woman described a situation in which she used the internet to understand why a laboratory result was abnormal: “That’s when I would go to Wikipedia [as a starting place] and I would check to see why my GFR [glomerular filtration rate] was low.”

The main difference between the information provided within the portal and on the internet, however, was that the portals did not generate suspicions of falsification. As a 72-year-old man said,

The patient portal is a reflection of what’s actually happened to you. The internet is a morass of good information and misinformation and it depends on your intellect or the space you’re in mentally as to how you interpret that.

When navigating the portal, participants noted fragmented and often perplexing information, but knew that the information within the portal was about them.

### Theme 4: Emphasizing the Importance of the Patient–Provider Relationship

#### Overview

Perhaps paradoxically, patients’ access to their personal health information via portals and an increased sense of independence have generated a greater emphasis on the value of patient–provider relationships. Although many participants voiced concerns about health care providers potentially withholding information as a means of protection or “sugar-coating” (as 1 participant has put it), participants still trusted and valued their professional advice. While the findings did not directly suggest that trust influenced patient portal use, they did highlight the importance of in-person interactions and having health care providers assist patients with interpreting information from the portals and other online sources. The subthemes for this category include trust and the essence of in-person interactions and the need for additional information.

#### Subtheme 4A: Trust and the Essence of In-person Interactions

Although participants appreciated having access to their personal health information, they did not want portals to replace the relationship they had with their oncologist, family doctor, or a nurse. The development of a trusting relationship between the patient and health provider was mainly attributed to in-person interactions. A 68-year-old woman stated, “I wouldn’t want it [the portal] to replace my relationship with my physician.” She continued, “I feel like I need to trust them. That relationship really matters and I’m not somebody who prefers to use technology for my relationships, I prefer it face-to-face.”

Participants described the importance of in-person interactions when receiving unpleasant news. A 72-year-old man rhetorically asked, “You should never have an internet message saying—‘you’ve got stomach cancer, report to your doctor’—that should never happen; that’s a human touch, right?” Comparably, a family caregiver said, “How it [a message] gets delivered, who you’re hearing it from, how you’re hearing it, makes a big difference in how you’re going to build your own frame of reference to go forward.” She continued, “They’re [health care providers] trained, they know how to deliver news like that and how to support people.”

#### Subtheme 4B: The Need for Additional Information

Most of the participants relied on their physician and nurses to provide them with necessary information, or to explain its significance, to understand and manage their medical condition. A 45-year-old woman shared: “My neutrophils, whenever I’m on my medication, is low. It doesn’t alarm me [when I see it in the portal] because I know my doctor’s seen it so if he was concerned about it then he would tell me.” Some of the participants did, however, recognize that their health care providers are also busy attending to other patients. A 60-year-old man said, “I found the doctors I was dealing with were also dealing with hundreds of other people.” Not having a health care provider available to interpret information significantly impacted the participants’ lives. A woman in her 20s who did not have a portal account shared her reality:

They’ll [health care providers] take weeks to get back to you and I think running on such high anxiety levels is simply something I can’t do. It really hinders every aspect of my life; I can’t function normally until I get the clear you know? It’s like debilitating fear.

Enlisting the help of formal supports, such as their oncologist, helped alleviate anxiety. A family caregiver shared, “She’s got a great family physician who will get all of her results and interpret them for her so when she actually talks to the oncologist she’s already in a state of receptivity, she’s more relaxed.” Similarly, another participant described her reaction to reading the word “metastases” on a radiology report within MyAHS Connect: “It made me very nervous.” She continued, “[but] now I know to ignore that because my doctor says, no, that’s not the case.” Participants acknowledged the importance of attending their medical appointments; for example, 1 woman stated, “That’s why we go to the specialist, to tie it all together.”

## Discussion

### Summary of Key Findings

The aim of this study was to explore the experiences of oncology patients or their family caregivers with electronic patient portals available in Alberta for health-related purposes. As far as we know, this is the first empirical study set in the unique context of a 2-portal system in Alberta, Canada, that illustrates how the tensions between the macro-level portal policy makers [22] are manifested in patient experiences with portal technology. At the time of the study, the provincial Government’s webpage with the access to MyHealth Records housed 2 portals. A provincial portal, My Personal Records (MPR; implemented by the Government per se), was available to adult Albertans, and most participants in our study used it. By contrast, a provincial health authority’s (AHS) clinical information system, Connect Care (EPIC) and its MyAHS Connect patient portal (MAC; known as MyChart in 2015-2019 during the pilot stage and implemented independently from the Government), had not been launched across Alberta’s oncology facilities. However, some oncology patients attending other clinics for concomitant health concerns might have had access to MyAHS Connect through those non-oncology facilities. One participant in our study used both portals.

One concern raised frequently by the participants was the lack of awareness of the portals in Alberta. Many pointed out that the portals were not well advertised. In fact, 3 participants who did not use the portals did not know they existed until enrolling in the study. Further, the overall terminological morass with portal names and an excessively complicated sign-up/authentication process are characteristics of the 2-portal context in the province. This influences the public perception and creates a barrier to portal adoption.

Our data do not permit robust comparison between the 2 portals (eg, webpage layout, navigation, filtering of test results); however, participants expressed frustration about the existing layout of My Personal Records, while MyAHS Connect was appreciated for providing access not only to laboratory tests but also to diagnostic images.

Findings of this study point to patients’ desire for transparency. Although portals and other digital platforms were considered as beneficial tools in accessing health information, these tools did not provide its users with direct information regarding their prognosis and future. Many of the participants used these tools as a means of triangulating or supplementing the information provided by their health care providers. Several participants wondered if their health care providers were withholding information from them as a means of protection; therefore, they used the portals and the internet to cross-check the information. Although the majority of participants felt that having access to health information enabled them to be more knowledgeable, prepared, and in control, some felt that having limited access to information prevented them from becoming active participants of their health. Moreover, many of the participants described how personal interactions had profound effects on the development of trusting patient–provider relationships and that they did not want portals or any other online tools to replace that.

Participants in our study did not regard searching for health information or using a portal in separation from their ongoing lives as people living with cancer. Related to the *technology-in-practice* perspective [26,27], we found that the portal joins the net of relations consisting of health care providers (especially oncologists and nurses), information, medical visits, diagnostic tests, prescribed drugs, family life, etc. The usefulness of portals (or not) is weighed by their ability to answer questions, link pieces of information, offer continuity through displaying comprehensive information, and make communication effortless. The organization of portal webpages and their content produce multiple and shifting effects such as increasing or alleviating anxiety, positioning a portal user as a tech-savvy or an “illiterate,” and enhancing or undermining trust in health services.

### Comparison With Other Literature

Supporting our findings, Kooij et al [12] noted a significant tension between the aims of protecting information privacy and facilitating portal uptake among end users. In the Netherlands, a portal sign up for patients that requires the use of the Government-issued unique digital identifier and a multistep authorization and verification is a notable barrier to portal uptake and use [12].

The evidence on the implementation and uptake of patient portals is unequivocal about the facilitating factors, such as creating awareness about the portal, easy sign-up process, intuitive navigation, explanation of medical terms, and the use of lay language [18]. Yet, all these facilitators were lacking at the time of the study.

Participants in our study emphasized the importance of the patient–provider relationship, a parallel finding to Alpert et al’s [17] study from the United States. In our study, the majority of the participants relied on their family doctor or oncologist to interpret information from the portals or the internet and to try to resolve feelings of uncertainty and distress. Similar findings were reported by Baudendistel et al’s [34] study in Germany, where health care providers shared their concerns of patients developing anxiety and uncertainty during the absence of professionals to interpret results presented within portals. Several participants preferred in-person interactions for communicating about their condition. The importance of communication in oncology is equally emphasized in several other American studies [9,35,36].

At the time of our study, participants lived with nonactive cancer, had infrequent diagnostic tests, and accessed the portal occasionally. With the exception of 1 person, they did not report situations when they viewed abnormal test results in real time, before their oncologist evaluated the results and had a chance to follow-up with them. By contrast, the research literature is replete with examples of concerns expressed by patients and health care providers about immediate result release. For example, the overwhelming majority of oncologists in an outpatient department at the Stanford Cancer Care Center felt that patient’s online access to abnormal results had negative consequences, but opinions were mixed for normal results [11]. Furthermore, half of the oncologists reported that sharing online results had worsened their communications with patients [11]. In another study, the timing of result release was identified by oncologists and nurses in a cancer care center in New York as particularly important for patients, as some results may indicate the recurrence or progression of disease, generating patient anxiety [37]. Physicians were clear about the necessity to quickly aid patients in interpreting test results to prevent or reduce anxiety [11].

Numerous studies suggest that electronic portals improve patient health outcomes [38,39]. Patient empowerment facilitated by the use of portals and other online tools is a recurring theme in the literature [3,9,38,40,41]. It is said that the provision of health information, especially laboratory results, allows patients to feel more involved in the management of their care, thereby empowering them [9,42]. Our findings complicate and add nuance to the aforementioned literature. Similar to findings reported in Ammenwerth et al [23], portals did not necessarily foster feelings of empowerment. Participants spoke of the challenges they encountered when attempting to become an informed and active member of the health care team. While access to health information allowed participants to prepare for their medical appointments and feel in control [17,43], many of them struggled to make sense of the fragmented information. Moreover, many of the participants discussed the need for access to information to self-manage in their daily life. Therefore, to foster feelings of empowerment, other conditions should be in place in addition to having access to one’s personal health information. It is possible that the language of empowerment is preferred by researchers, but people living with cancer seem to describe their experiences in other ways.

### Recommendations for Research

Contrary to some existing research, in our study, participants who used a portal did not describe feelings of empowerment. We wondered: do portals and other online tools actually foster feelings of empowerment or does this notion stem from the development of knowledge about one’s condition and health-visit preparation skills? The interchangeable use of the terms *engagement* and *empowerment* has further added to the complexity of measuring this concept [42]. Future research might explore both engagement and empowerment and clearly define how these terms are understood.

Further, ethnographic fieldwork is promising for understanding why portals are used or abandoned by patients and involves examining whether and how patients use health technology in daily life, what practical arrangements (consisting of people and things) they create to support living with chronic health conditions, and how technology can support what Jeannette Pols calls a *good life* for patients [44]. Talking about *good* life with technology, Pols, a social scientist, philosopher, and health care researcher, means that the new health technology (eg, a portal) is not inherently good. Its effects and outcomes are not predetermined but instead are produced as the result of interactions among various human and nonhuman elements in everyday life. This draws attention to particularities [45], and to the necessity for accommodations, the ability to undertake and undergo small changes and adjustments from/by technological systems, humans, health care practices, and policies. What Pols might ask of portal implementers, policy makers, health care organizations, and researchers interested in the success of eHealth tools is to—amidst the focus on health care standardization, “generalizable outcomes,” “universal values,” and “general trends” [45]—make space to attend to particularities of patients’ lives to understand what arrangements make a portal valuable versus meaningless.

A noteworthy finding of the study was that some participants used the portal and the internet to counter the lack of transparency perceived in health care. Future studies can explore how trust can be developed and sustained within online environments. Transparency is seldom discussed in health care despite being a common concern and potential ethical issue that directly impacts patient care [46]. Full disclosure of information may promote better quality care, augment trust, and promote better health outcomes [47].

Future research could also examine portal platforms and compare them across Canada, as some provinces work with different vendors and develop their own portals. Comparing portal implementation across the country could assist with the identification of best practices and help guide improvement strategies to reduce costs and maximize benefits.

Once Connect Care is launched within Alberta cancer care facilities and patients receive access to MyAHS Connect (MAC), it will be essential to understand patients’ experiences with the 2-portal terrain as well as health professionals’ perspectives working within the context of oncology care. Some areas that will need to be considered include access to the portal (ensuring an easier sign-up process) and ways to balance transparency with the potential psychological impact of information that is distressing, unclear, or can be misinterpreted. With increased portal use and the expansion of the potential information that can be accessed by both the patient and their families via a proxy access, further questions arise. These questions also highlight the ease of use and the security of the data.

### Recommendations for Practice

One key recommendation is to improve public awareness and health care providers’ awareness about portals and their ability to promote them. Further, developing an education program (eg, video tutorials and posters) can facilitate portal uptake. Health care providers also require portal training, as it may allow them to assist patients who require further support with accessing supplementary resources and navigating portals. Education programs aiming to increase citizens’ digital and health literacy may assist patients to develop confidence, critically analyze health information, and allow them to make informed decisions that optimize their health [48]. Health care providers are at the forefront of patient education and might be in the optimal positions to tailor education sessions to individual capacity [49]; however, health care providers require organizational support and would need to co-design educational materials with patients and family members.

Our study did not include perspectives of oncology service providers; however, it is well known that the collection, storage, and analysis of patient-reported quality of life and outcome measures is an ongoing process in the oncology context. Patient portals provide a convenient venue to support these organizational goals, making it easier for patients to complete before- and after-visit questionnaires. The success of this undertaking depends on patient’s uptake of the portal technology. Our findings indicate that even highly educated and literate individuals with computer skills might be deterred from the difficult-to-navigate portals containing fragmented information.

### Recommendations for Policy

A patient-friendly version of the portal with a simpler interface, and one that is designed with an understanding of *how* patients use information, is needed. However, explaining the significance of laboratory values and providing direction on what to do after being informed about an abnormal result lie beyond the portal’s affordances; it is the role of the clinician. Portal policies should be developed with the appreciation of the role of clinicians, who often need to mediate between the patient and the portal.

It will be interesting to observe how the Alberta Government’s My Personal Record and the health authority’s MyAHS Connect coevolve and how this process shapes experiences of portal users. Another important consideration is the timing of releasing test results into the portals. Many oncology patients prefer discussing the results with the oncologist first to prevent feelings of distress. Lastly, an essential recommendation for practice and policy is that portals cannot streamline or replace the patient–provider relationship, as this relationship can provide both trusting and individualized care [50].

### Limitations

There are a few limitations to this study. All participants spoke English as their primary language; therefore, this study did not account for challenges that may have been faced by individuals who speak English as an additional language, or who are unable to speak English. Further, our convenience sample comprised individuals from the Alberta Patient and Family Advisory Network for oncology. These tend to be well-educated individuals (often former health care professionals) who regularly use computers and the internet and are active participants in managing their health. Lastly, our recruitment relied exclusively on email invitation (with 1 reminder). This approach may have excluded individuals and groups who do not use computers and who, by extension, will likely not be able to use portals.

The strength of this study was a sample comprising individuals of diverse age, from the 20s to the late 70s. Further, patient portals are new to Alberta, Canada, and it is informative to learn from the experiences of early adopters. The detailed description of portal features and the context of portal implementation provided earlier in this paper will help readers judge the degree of transferability of our findings. Indeed, we want to stress that the differences in portal features and design across jurisdictions should be taken into consideration in research on portals.

### Conclusion

In Canada, the objective of using eHealth is to encourage Canadians to live healthier by offering online tools that securely connect its users with valid, up-to-date health information to augment understanding and management of personal health [2]. With the growth of cancer diagnoses today, patient portals are becoming more desirable to strengthen the coordination of care for oncology patients [11]. Although literature foregrounds the benefits that portals can offer patients, the findings of this study suggest that more effort is needed to move from the portal deployment to making it an integral tool in the lives of people living with cancer. It is noteworthy that patient portals cannot replace the patient–provider relationship, but rather serve as an additional means of accessing information and assisting oncology patients to cope with their condition.

## Appendix

Multimedia Appendix 1 Comparison of main patient portal features in Alberta, Canada, as of Fall 2020.

## Abbreviations

AHS: Alberta Health Services
EMR: electronic medical record
MAC: MyAHS Connect (AHS’ Connect Care portal)
MADI: MyAlberta Digital ID
MPR: My Personal Records (Alberta Health [government] portal)
UHN: University Health Network
