# Supplementary material for: Oncology Patients’ Experiences With Novel Electronic Patient Portals to Support Care and Treatment: Qualitative Study With Early Users and Nonusers of Portals in Alberta, Canada
Source: JMIR Cancer. 2021 Nov 24;7(4):e32609. doi: 10.2196/32609 (PMC8663539; doi:10.2196/32609)
Supplement: Multimedia Appendix 1 [file cancer_v7i4e32609_app1.doc]

**Appendix**

**Comparison of main patient portal features in Alberta, Canada, as of Fall 2020**

| Patient portal functionalities  (##1-5 adapted from Ammenwerth et al. 2021) | Portal implementer: the integrated provincial health authority Alberta Health Services  (Portal provider: Epic Systems Corporation) | | Portal implementer:  Provincial Government’s branch Alberta Health (Provider: Telus) |
| --- | --- | --- | --- |
|  | **MyChart***  (Pilot in 2016-2019) | **MyAHS Connect ****  (MyChart was renamed following the ConnectCare launch in Nov 2019) | **My Personal Records****  (Initially launched as MyHealth Records in March 2019; in early 2020, MHR became a single point of entry for both My Personal Records—a government’s portal per se—and for MyAHS Connect) |
| 1. **Access personal health data** | | | |
| View lab results | YES  Several results released in real time (hourly) e.g., hematology. Other results released after 10-day delay (or earlier if provider does so manually) e.g., pathology reports, genetic testing, cancer-specific tests. | YES  Most test results are released as soon as they are available. Other results take several days to weeks. Results may also be delayed if a doctor feels a face-to-face explanation is required. Some results are not released into MyAHS Connect. | YES  Patients are able to view only 50+ common lab test results, as soon as they are available – usually within 24h. |
| View diagnostic imaging reports | YES  Released after 10-day delay (or earlier if provider does so manually) | YES  The 5-day delay of the release of diagnostic imaging, cardiology and endoscopy reports | NO |
| View medications | YES | YES | YES |
| View immunizations | YES | YES | YES |
| View appointment dates in AHS facilities  (*Note: most primary care, community care, family GPs are not part of AHS)* | YES | YES | NO |
| 1. **Communicate** | | | |
| Secure bidirectional messaging between patient and AHS care team for non-urgent questions *(Note: most primary care, community care, family GPs are not part of AHS)* | YES | YES | Initially NO.  Messaging added in May 2020; requires health providers and patients to use the same messaging tool provided by the third party Brightsquid Secure-Mail. Patients can create their own Secure-Mail account in MPR to communicate virtually with community healthcare providers |
| Video visits | Not enabled | YES (via Zoom) but is currently limited | NO |
| 1. **Request** | | | |
| Book and modify AHS appointments | Limited availability based on piloting clinic’s decision | YES but depends on provider’s set up and is currently limited | NO |
| 1. **Share (patient self-documentation)** | | | |
| Complete pre-visit questionnaires for AHS appointments | Planned but not used | YES but depends on provider’s set up and is currently limited | NO |
| Enter and track home data (e.g., blood pressure, glucose) | Not enabled | YES  “share smartphone and medical device data (Apple Health, Google Fit, Fitbit, and Withings compatible devices) with AHS and partner care teams” | YES  upload and track info from personal health devices e.g., blood pressure monitors, blood glucose meters, and fitness trackers that are supported by My Personal Records |
| 1. **Educate** | | | |
| Access general educational health-related information | ? | YES  “some direct links to the MyHealth.Alberta.ca website” for info about tests | YES  Clicking on a link in a test result will bring up a clinical abstract about that test from  MyHealth.Alberta.ca website |
| 1. **Proxy access** | | | |
| Adult to child proxy  Adult to adult proxy | YES | YES but the process is complicated.  E.g., parents or guardians of AHS patients under 18 years can request access to the minor patient’s MyAHS Connect record via a proxy access after it is enabled by an appropriate AHS provider.  Both a proxy and a family member must each have:  a) their own MADI and MHR accounts;  b) their own MyAHS Connect accounts. | NO  MPR only allows a user (i.e., an Albertan at least 14 years old) to share their record with someone they trust, but no proxy access in possible.  For those youth 14 y.o. and over who have MHR and decide to provide another person access to their MPR, there is a sharing feature to invite and electronically ‘share’ some or all of their MHR record. The user is the only one that has control of this feature. |
| 1. **Mobile options** (tablet, smartphone) | | | |
|  | YES  "MyChart" for iOS and "MyChart" for Android | YES  "MyChart" for iOS and "MyChart" for Android | YES |
| Sources: 1 | 2, 3, 4 | 5, 6, 7 | 7, 8, 9 |

*Features made available and taken up in the first 5 piloting clinics were: view appointments, test results, and medications; securely message health care providers through a computer or an app on a phone or tablet. Only 1 clinic out of 5 implemented the MyChart web-based booking and canceling appointment’s function [2]. Adult-to-child proxy access was implemented in 1 pediatric clinic that piloted MyChart in 2018-2019 (Petrovskaya O., unpublished data).

** These portals are constantly evolving. Throughout 2021, both MPR and MyAHS Connect expanded and aligned the range of lab results released. About 95% of the most commonly ordered results including hospital and ER results are released immediately. As of July 2021, diagnostic imaging, cardiology and endoscopy reports are still not available in MPR but are available to AHS patients via MyAHS Connect after a 5-day delay. Alberta Health announced that all remaining test results available in Netcare EMR, including complex results in cytology, pathology, genetics will be released immediately to MPR by the end of September 2021.

Sources:

1. Ammenwerth E, Neyer S, Hörbst A, Mueller G, Siebert U, Schnell-Inderst P. Adult patient access to electronic health records. Cochrane Database Sys Rev 2021 Feb; 2: CD012707. [doi: 10.1002/14651858.CD012707.pub2]
2. Avdagovska M, Ballermann M, Olson K, Graham T, Menon D, Stafinski T. Patient portal implementation and uptake: qualitative comparative case study. J Med Internet Res 2020 Jul;22(7): e18973. [doi: 10.2196/18973]
3. Avdagovska M, Stafinski T, Ballermann M, Menon D, Olson K, Paul P. Tracing the decisions that shaped the development of MyChart, an electronic patient portal in Alberta, Canada: historical research study. J Med Internet Res 2020 May;22(5): e17505. [doi: 10.2196/17505]
4. Graham TAD, Ali S, Avdagovska M, Ballermann M. Effects of a web-based patient portal on patient satisfaction and missed appointment rates: survey study. Journal of Medical Internet Research 2020; 22(5). [doi: 10.2196/17955]
5. <https://myahsconnect.albertahealthservices.ca/MyChartPRD/Authentication/Login?mode=stdfile&option=faq>
6. <https://www.albertahealthservices.ca/cis/page15448.aspx>
7. [https://myhealth.alberta.ca/UAM/MyHealthRecords/Pages/MHRProviders.aspx#labresults](https://myhealth.alberta.ca/UAM/MyHealthRecords/Pages/MHRProviders.aspx#labresults)
8. <https://www.canhealth.com/2020/07/29/alberta-integrates-secure-messaging-from-brightsquid/>
9. <https://www.albertanetcare.ca/documents/MHR-SecureMail-GeneralFAQs.pdf>
